# Entropy-Based Machine Learning Model for Fast Diagnosis and Monitoring of Parkinson’s Disease

**DOI:** 10.3390/s23208609

**Published:** 2023-10-20

**Authors:** Maksim Belyaev, Murugappan Murugappan, Andrei Velichko, Dmitry Korzun

**Affiliations:** 1 Institute of Physics and Technology, Petrozavodsk State University, 185910 Petrozavodsk, Russia; velichko@petrsu.ru; 2 Intelligent Signal Processing (ISP) Research Lab, Department of Electronics and Communication Engineering, Kuwait College of Science and Technology, Block 4, Kuwait City 13133, Kuwait; m.murugappan@kcst.edu.kw; 3 Department of Electronics and Communication Engineering, Faculty of Engineering, Vels Institute of Sciences, Technology, and Advanced Studies, Chennai 600117, India; 4 Centre of Excellence for Unmanned Aerial Systems (CoEUAS), Universiti Malaysia Perlis, Arau 02600, Perlis, Malaysia; 5 Department of Computer Science, Institute of Mathematics and Information Technology, Petrozavodsk State University, 185910 Petrozavodsk, Russia; dkorzun@cs.karelia.ru

**Keywords:** Parkinson’s disease, EEG, diagnosis, entropy, machine learning, monitoring, smart IoT environment, edge device, human resilience

## Abstract

This study presents the concept of a computationally efficient machine learning (ML) model for diagnosing and monitoring Parkinson’s disease (PD) using rest-state EEG signals (rs-EEG) from 20 PD subjects and 20 normal control (NC) subjects at a sampling rate of 128 Hz. Based on the comparative analysis of the effectiveness of entropy calculation methods, fuzzy entropy showed the best results in diagnosing and monitoring PD using rs-EEG, with classification accuracy (*A*_RKF_) of ~99.9%. The most important frequency range of rs-EEG for PD-based diagnostics lies in the range of 0–4 Hz, and the most informative signals were mainly received from the right hemisphere of the head. It was also found that *A*_RKF_ significantly decreased as the length of rs-EEG segments decreased from 1000 to 150 samples. Using a procedure for selecting the most informative features, it was possible to reduce the computational costs of classification by 11 times, while maintaining an *A*_RKF_ ~99.9%. The proposed method can be used in the healthcare internet of things (H-IoT), where low-performance edge devices can implement ML sensors to enhance human resilience to PD.

## 1. Introduction

By 2030, experts predict that every sixth person on Earth will be over 60 years of age due to an increasing life expectancy [1]. It is estimated that 1.4 billion people will be over 60 by 2050. Age-related neurodegenerative diseases are a major risk factor for mortality and morbidity caused by neurodegenerative diseases [2,3,4]. The symptoms of neurodegenerative disease may begin as early as middle age [5], followed by overt signs and symptoms. By diagnosing and treating patients early, irreversible damage to the nervous system can be reduced, improving their quality of life and length of life.

In addition to diagnostics, a personalized approach to neurodegenerative disease treatment using IoT-enabled environments is essential to improving patients’ quality of life [5,6,7], such as smart homes and healthcare [8], smart spaces for mHealth applications [9], and smart healthcare [8]. The healthcare IoT (H-IoT) [10] is also known as IoMT [11] and is one of the most efficient tools for this purpose. The key point is that a sensor (within an IoT edge device) participates in making the device smart. In particular, machine learning (ML) methods can be used to analyze the sensed data for diagnosis, e.g., see our concept of an ML sensor for diagnosing COVID-19 [12]. The problem is that IoT edge devices are of low performance, and new effective ML algorithms are required.

Presently, some methods are available for detecting neurodegenerative diseases. Using data collected from question assessments [13], blood biomarkers [14], eye-tracking parameters [15], kinematic gait parameters [16], electroencephalograms (EEG) [17], and other tests, conclusions are drawn regarding the presence or high risk of developing PD.

Among the disease indicators presented, EEG is one of the most promising because of its non-invasive nature, wide distribution, low cost, and ability to be integrated into the internet of things (IoT) [18]. In addition to diagnosing diseases, the use of portable personal devices for recording EEG can be used to continuously monitor the patient’s current condition and the effectiveness of the selected treatment method outside of the medical institution through the development of intelligent devices with IoT technology. Usually, EEG signals can be analyzed in time, frequency, and time–frequency domains [19]. A time–frequency analysis can be performed by applying a short-time Fourier transform [20] and wavelet transform [21] to examine the local temporal effects that occur under specific bands of EEG frequencies. Generally, five different frequency bands are investigated in EEG signals depending on their application. These bands are delta (0–4 Hz), theta (4–8 Hz), alpha (8–13 Hz), beta (13–30 Hz), and gamma (30–49 Hz) [22].

Electrical signals from the brain are highly non-stationary and complex. They are susceptible to disturbances caused by external and internal noises. To characterize EEG signal behavior in each class, different types of features are needed, such as statistical, spectral, and entropy characteristics [23,24,25]. Various machine learning methods are then used to determine the presence and type of neurodegenerative diseases [26,27,28,29,30,31]: artificial neural network (ANN), probabilistic neural network (PNN), support vector machine (SVM), neural network (NN) including deep learning neural network (DNN), decision tree (DT), random forest (RF), Bayesian model (NB), k-nearest neighbor method (KNN), etc.

Rest-state EEG signals can be used to detect a variety of neurodegenerative diseases, including Alzheimer’s disease [32], Parkinson’s disease (PD) [28,29,30,31,33,34,35], frontotemporal dementia [36], dementia with Lewy bodies [37], and epilepsy [38]. In the field of neurodegenerative diseases, Parkinson’s disease is one of the most studied. As a result of Parkinson’s disease, a person will have impaired motor functions (slowness of movement, tremors, rigidity, and loss of balance) and impaired non-motor functions (decreased cognitive functions, mental disorders, sleep disturbances, pain, and sensory problems) [39]. As part of PD diagnostics, the EEG signal of the patient is compared to the EEG signal of a healthy control group. Depending on the metric, changes can be determined either by comparing signals directly [34] or by quantifying them using entropy metrics, spectral power metrics, cross-correlation metrics, statistical values, etc., [28,29,30,31,33,35]. In [34], convolutional neural networks are used to classify PD using a deep learning approach, in which the elements of the filtered signal are fed into the neural network as input. A classification accuracy of 88.2% was achieved by this approach. However, using calculated features usually yields better results. A principal component analysis of the filtered signal, correlation coefficients, and linear predictive coefficients is used to calculate features for the SVM classifier [35] that achieved a maximum mean classification rate of 99.1% in diagnosing PD. Due to the fact that EEG changes can occur at certain frequency ranges (corresponding to alpha, beta, gamma, theta, and delta waves), translating the signal from the time domain (Fourier transform) or frequency–time domain (wavelet transform) is a common method of analyzing EEG signals. In [30], spectral features (such as wavelet coherence and relative wavelet energy) were used to detect PD-related dementia, AD, and a control group. Spectral energy differences were found between the control group and the rest of the patients at both low and high frequencies. They were able to determine PD with an accuracy of 79.1% and AD with an accuracy of 81.2% using linear discriminant analysis. By using the tunable Q wavelet transform, statistical signal metrics were extracted from the frequency subbands of the rest-state EEG signal (minimum, Hurst exponent, Higuchi fractal dimension, Hjorth complexity, mobility). There are four types of classifiers used to distinguish PD from healthy controls (ANN, SVM, KNN, and RF) based on the above-mentioned statistical features. As a result, the mean classification accuracy for healthy controls and PD patients (with or without medical treatment) was 96.1% and 97.7%, respectively.

Several works have focused on the differences in the entropy of signals in different frequency ranges between patients with PD and the control group [28,31,33]. In [33], relative spectral powers and wavelet packet entropy were used to identify PD. Although entropy features allow for better separation of two classes, relative spectral power (especially in the beta band) can also be useful. Higher-order spectral features, like bispectral entropies and mean magnitude, were used for PD diagnosis [28] based on five different types of classification algorithms such as DT, KNN, NB, PNN, and SVM. The SVM classifier reported a maximum mean accuracy of 99.6% compared to other classifiers in diagnosing PD. The authors of [31] used the KNN and SVM classifiers to diagnose PD based on energy and entropy features extracted from reconstructed wavelet signals. Accordingly, KNN and SVM classifiers achieved 99.5% and 99.9% mean accuracy, respectively.

Although the presented results prove a high classification accuracy (more than 99%), most of the approaches used to calculate features are limited. Also, the hyperparameters used when calculating entropy can significantly affect the calculation result. To obtain high accuracy, a number of studies have used many features [30,31,33,34], which complicates the implementation of these methods in low-performance IoT devices. This paper attempts to address these deficiencies by comparing various entropy methods, carefully selecting their parameters, and analyzing EEG signal frequency ranges for diagnosing PD. By analyzing EEG data collected from normal control (NC) and Parkinson’s disease (PD) patients using wireless Emotiv EPOC headsets, we have developed a novel method for detecting PD which can be used in a smart IoT environment to enhance human resilience to PD.

The major contributions of this paper are:

- A comparative analysis of the effectiveness of various methods for calculating entropy for identifying PD was carried out;
- The most significant frequency ranges and EEG channels were identified, as well as their combinations;
- A study was conducted to reduce computational costs by selecting the most significant features and reducing the length of the EEG segments analyzed;
- A method of monitoring a patient’s condition based on entropy values was developed;
- We propose a machine learning model for monitoring the health status of Parkinson’s patients using an IoT environment based on low-performance sensors.

The remainder of this paper is organized as follows. Section 2 provides an overview of the datasets, proposed methods, and performance evaluation. Section 3 presents a comparison of classification accuracy using different EEG channels and frequency bands. Section 4 describes options for optimizing the classification model by reducing the length of the EEG segment and the number of features. Section 5 outlines our further research on the smart IoT environment concept for patient health monitoring and enhancing human resilience. Section 6 summarizes the key findings and limitations of our study.

## 2. Materials and Methods

### 2.1. Dataset

**Figure 1:**
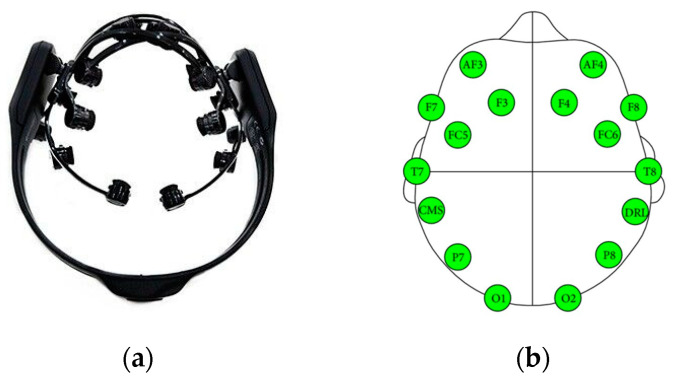
Emotiv EPOC wireless headset (**a**). Location of the electrodes on the head (**b**) [43].

This study was conducted using an EEG dataset consisting of 20 patients with Parkinson’s disease and 20 age-matched normal control subjects without a history of psychological disorders or neurological disorders. This dataset was collected at the Hospital Universiti Kebangsaan Malaysia in Malaysia. The entire data acquisition protocol at the Hospital Universiti Kebangsaan Malaysia was approved by the Institutional Ethical Review Board Committee as part of the hospital’s ethical review process. An Emotiv EPOC wireless headset with a total of 14 channels (Figure 1a) was used for recording EEG signals from both NCs and PDs in the rest-state condition with the eyes closed for a period of 5 min during this study. In accordance with the international standard 10–20 system, the 14 channels (AF3, F7, F3, FC5, T7, P7, O1, O2, P8, T8, FC6, F4, F8, AF4) were placed on the subject’s scalp (Figure 1b). With a sampling rate of 128 Hz, the data collected for each of the channels were converted into digital signals. Using the Hoehn and Yahr scales, a total of seven patients were classified as having Parkinson’s disease stage III, eleven patients as having Parkinson’s disease stage II, and two patients as having Parkinson’s disease stage I. A complete description of the dataset, acquisition, and preprocessing of the dataset can be found in [40,41,42].

**Figure 2:**
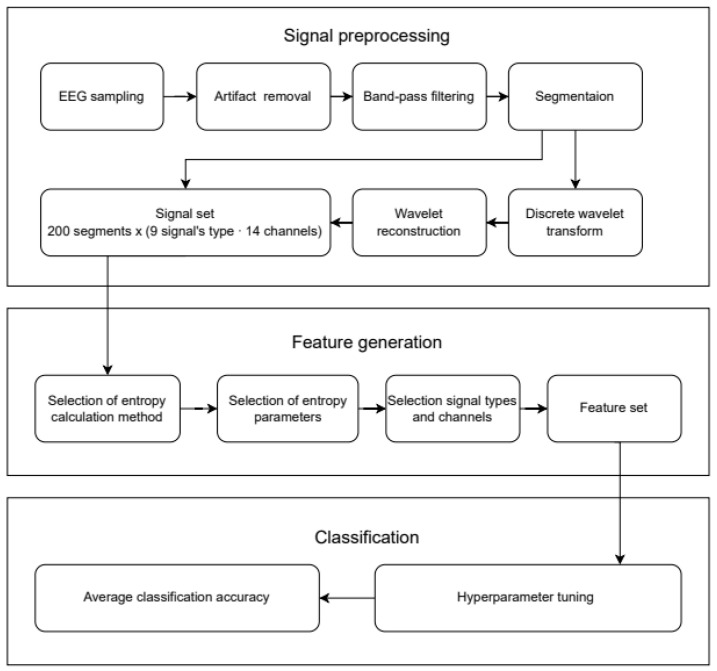
The workflow diagram of the proposed classification method.

Figure 2 shows the workflow diagram of the proposed classification method. It consists of three separate steps: signal preprocessing (Section 2.2), feature generation (Section 2.3) and classification (Section 2.4).

### 2.2. Signal Preprocessing

Considering the wide spectral range of EEG signals (0–64 Hz) and the fact that most brain activity information is contained in relatively narrow frequency subranges [44,45], it is possible that the entropy of the original signal gives a poor indication of its separation capability. Filtering the initial data and decomposing the signal into separate frequencies using the wavelet transform can increase EEG signals’ information content. A fifth-order Butterworth filter with a cut-off frequency of 0.5–32 Hz was applied to all acquired signals to remove low- and high-frequency noise, while amplitude thresholding of ±85 µV was applied to remove artifacts (eye blinking, eyeball rotation, and eye movements) during the acquisition process. Since the number of patients was relatively small, each EEG record was divided into 5 non-overlapping segments, each of which represented an independent observation within the framework of this study. The duration of all segments was the same and varied from 150 (~1.2 s) to 1000 (~7.8 s) samples. A discrete wavelet transform (DWT) was performed on the signal using the db4 wavelet [46]. After decomposing into wavelet approximation coefficients (A1–A4) and details (D1–D4), each of them was utilized to reconstruct the signals, with each signal (cA1–cA4 and cD1–cD4) being reconstructed with only one of the coefficients. A similar method was used in [31]; however, a different frequency band was chosen.

Using the original dataset, 9 variants of different signal types were obtained:

- Original (O) signal; frequency ranges: (0–64 Hz);
- Signals reconstructed based on approximation coefficients (cA1–cA4); frequency ranges: (cA1 (0–32 Hz), cA2 (0–16 Hz), cA3 (0–8 Hz), cA4 (0–4 Hz));
- Signals reconstructed based on detail coefficients (cD1–cD4); frequency ranges: (cD1 (32–64 Hz), cD2 (16–32 Hz), cD3 (8–16 Hz), cD4 (4–8 Hz)).

### 2.3. Feature Generation

Entropy features were calculated from EEG signals after applying DWT and concatenated to form the feature vector for each class (NC and PD). Later, these feature vectors were used in classifying patients using different machine learning methods. This entropy model comprises several features such as singular value decomposition entropy (SVDEn) [47], permutation entropy (PermEn) [48], sample entropy (SampEn) [49], cosine similarity entropy (CoSiEn) [50], fuzzy entropy (FuzzyEn) [51], phase entropy (PhaseEn) [52], and attention entropy (AttnEn) [53]. A method for calculating entropy was implemented using the EntropyHub (version 0.2) [54] software package, except for SVDEn and PermEn. The Antropy (version 0.1.6) [55] software package was used to calculate SVDEn and PermEn. The range of hyperparameters used for computing each type of entropy is shown in Table 1. There are no hyperparameters associated with AttnEn.

Below are descriptions of these methods for calculating entropy (Section 2.3.1, Section 2.3.2, Section 2.3.3, Section 2.3.4, Section 2.3.5, Section 2.3.6 and Section 2.3.7).

#### 2.3.1. SVDEn

To calculate SVDEn for a time series *X* = [*x*_1_, *x*_2_, … *x_i_*, … *x_N_*] of length *N*, an embedding matrix *A* is created as follows:

(1) $$\begin{array}{l} a(i)=[x_{i},x_{i+delay},\ldots ,x_{i+(m-1)\cdot delay}]\\ A={\left[a(1),a(2),\ldots ,a(N-(m-1)\cdot delay)\right]}^{T} \end{array}$$

where *m*—length of the embedding dimension and *delay*—time series sample bias.

Singular value decomposition is the factorization of matrix *A* into the product:

(2) $$A=USV^{T}$$

Matrix *U* contains the left singular vectors of *A*, and matrix *V* contains the right singular vectors. Matrix *S* is always diagonal, and its coefficients are non-negative real numbers *λ*_1_, …, *λ_k_*, located on the main diagonal of the matrix, which are called singular values.

The dispersion of singular values *λ_k_* also provides an indication of the complexity of signal dynamics [47]. Singular values can be normalized as:

(3) $${\bar{\lambda }}_{k}=\frac{\lambda_{k}}{\sum \lambda_{k}}$$

Singular value decomposition entropy is defined with the Shannon formula applied to the elements of singular values of the matrix, and calculated as follows [47]:

(4) $$\mathrm{SVDEn}=-\sum {\bar{\lambda }}_{k}\cdot \ln{\bar{\lambda }}_{k}$$

After that, the SVDEn values are normalized in the range from 0 to 1:

(5) $$\mathrm{SVDEn}=\frac{\mathrm{SVDEn}}{\log_{2}m}$$

#### 2.3.2. PermEn

PermEn is a complexity measure for time series based on the comparison of neighboring values. The permutation entropy PermEn of a one-dimensional data series *X* is:

(6) $$\mathrm{PermEn}=-\sum p_{i}\cdot \log_{2}p_{i}$$

where *p_i_*—the frequency of occurrence of the *i*-th permutation in embedded matrix *A*, which is defined in the same way as (1).

After that, PermEn values are normalized in the range from 0 to 1:

(7) $$\mathrm{PermEn}=\frac{\mathrm{PermEn}}{\log_{2}m!}$$

#### 2.3.3. SampEn

The SampEn calculation of time series *X* = [*x*_1_, *x*_2_, … *x_N_*] of length *N* contains several stages. First, the series is divided into template vector $X_{i}^{m}$ = [*x_i_*, *x_i_*_+1_, … *x_i_*_+*m*−1_] of length *m* (*m* < *N*). Then, the number *C* (*m*, *r*) of pairs of vectors $X_{i}^{m}$ and $X_{j}^{m}$ (*i* ≠ *j*) for which the Chebyshev distance ChebDist[$X_{i}^{m}$, $X_{j}^{m}$] does not exceed *r* is calculated.

SampEn for one-dimensional data series *X* is defined as:

(8) $$\mathrm{SampEn}=-\ln(\frac{C(m+1,r)}{C(m,r)})$$

#### 2.3.4. CoSiEn

The CoSiEn calculation of time series *X* = [*x*_1_, *x*_2_, … *x_N_*] of length *N* contains several stages. First, the series is divided into template vector $X_{i}^{m}$ = [*x_i_*, *x_i_*_+1_, … *x_i_*_+*m*−1_] of length m (*m* < *N*). Then, the number *B* (*m*, *r*) of pairs of vectors $X_{i}^{m}$ and $X_{j}^{m}$ (*i* ≠ *j*) for which the angular distance *AngDist*[$X_{i}^{m}$, $X_{j}^{m}$] does not exceed *r* is calculated.

Angular distance between two vectors is calculated as follows:

(9) $$AngDist=\frac{1}{\pi }\cdot \cos{\left(\frac{X_{i}^{m}\cdot X_{j}^{m}}{\left|X_{i}^{m}||X_{j}^{m}\right|}\right)}^{-1}$$

CoSiEn for one-dimensional data series *X* is defined as:

(10) $$\mathrm{CoS}\mathrm{iEn}=-\left[B(m,r)\cdot \log_{2}B(m,r)+(1-B(m,r))\cdot \log_{2}(1-B(m,r))\right]$$

#### 2.3.5. FuzzyEn

For a vector of time series *T* of length *N*, it is possible to compose *N* – *m* + 1 vectors $X_{i}^{m}$ of length m, consisting of normalized successive segments of the original series *T*. The normalization procedure consists of subtracting $T_{i}^{avg}$ from each element of the series:

(11) $$X_{i}^{m}=\{x_{i},x_{i+1},\ldots x_{i+m-1}\}=\{T_{i},T_{i+1},\ldots ,T_{i+m-1}\}-T_{i}^{avg}$$

where *i* = 1…*N* – *m* + 1 and $T_{i}^{avg}$ is calculated as follows:

(12) $$T_{i}^{avg}=\frac{1}{m}\sum_{j=0}^{m-1}T_{i+j}$$

For any pair of vectors $X_{i}^{m}$ and $X_{i}^{m}$ (*i* ≠ *j*), one can determine the distance $d_{ij}^{m}$ between them equal to the maximum absolute difference between the vector components:

(13) $$d_{ij}^{m}=\underset{k\in (0,m-1)}{\max}\left|x_{i+k}-x_{j+k}\right|$$

The similarity between vectors is determined using the fuzzy function $D_{ij}^{m}$:

(14) $$D_{ij}^{m}=\exp(-\frac{{\left(d_{ij}^{m}\right)}^{r_{2}}}{r})$$

The FuzzyEn entropy value is calculated based on the average similarity of vectors. For a finite series T it can be expressed as:

(15) $$\mathrm{FuzzyEn}=\ln(\phi^{m})-\ln(\phi^{m+1})$$

where the function *ϕ^m^* is expressed through:

(16) $$\phi^{m}=\frac{1}{N-m}\sum_{i}^{N-m}\left(\frac{1}{N-m-1}\sum_{j=1,j\neq i}^{N-m}D_{ij}^{m}\right)$$

#### 2.3.6. PhaseEn

In order to calculate PhaseEn of time series *X* = [*x*_1_, *x*_2_, … *x_N_*] of length *N*, it is necessary to first construct vectors *Y* and *W*, which are the coordinates of the points on the second-order difference plot, defined as follows:

(17) $$\begin{array}{l} Y=[x_{3}-x_{2},x_{4}-x_{3},\ldots ,x_{N}-x_{N-1}]\\ W=[x_{2}-x_{1},x_{3}-x_{2},\ldots ,x_{N-1}-x_{N-2}] \end{array}$$

Then, a vector containing the slope angles of each point (in the range of 0–2π) is calculated as follows:

(18) $$\theta =\tan^{-1}(\frac{Y}{W})$$

Then, the entire range (2π) is divided into *K* equal sectors, for each of which the total slope angle *S_i_* (*i* = 1…*K*) is calculated:

(19) $$S_{i}=\sum_{j=1}^{N}\theta_{j},if\;\theta_{j}\in \left[\frac{(i-1)\cdot 2\pi }{K},\frac{i\cdot 2\pi }{K}\right]$$

After that, probability distribution *p_i_* is calculated for each of the *K* sectors:

(20) $$p_{i}=\frac{S_{i}}{\sum_{j=1}^{K}S_{j}}$$

PhaseEn is computed as:

(21) $$\mathrm{PhaseEn}=-\frac{1}{\log K}\sum_{i=1}^{K}p_{i}\cdot \log p_{i}$$

#### 2.3.7. AttnEn

The AttnEn calculation of time series *X* = [*x*_1_, *x*_2_, … *x_N_*] of length *N* contains several stages. First, it is necessary to calculate the positions of local minima and maxima within the time series. By local minimum, we mean point x_i_ for which the inequalities *x_i_* < *x_i_*_−1_ and *x_i_* < *x_i_*_+1_ hold, and by local maximum, we mean point *x_j_* for which the inequalities *x_j_* > *x_j_*_−1_ and *x_j_* > *x_j_*_+1_ hold. Then, the intervals between two successive peak points (minima and maxima) are calculated. In this case, 4 variants of such intervals are considered: between two maximums (*I*_max-max_), between two minimums (*I*_min-min_), between the maximum and the subsequent minimum (*I*_max-min_), between the minimum and the subsequent maximum (*I*_min-max_).

After calculating 4 sets of intervals (*I*_max-max_, *I*_min-min_, *I*_max-min_, *I*_min-max_) for each set, the frequency of occurrence of each interval within the set is calculated, on the basis of which Shannon entropy values are calculated (ShEn_max-max_, ShEn_min-min_, ShEn_max-min_, ShEn_min-max_). The AttnEn value is the average of these entropies: AttnEn = (ShEn_max-max_ + ShEn_min-min_ + ShEn_max-min_ + ShEn_min-max_)/4.

### 2.4. Assessment of Classification Accuracy

The accuracy of the classifications was assessed using support vector classifiers (SVCs) implemented using scikit-learn. Two stages were involved in the classification accuracy assessment. In the first step, hyperparameters were selected by means of repeated K-fold cross-validation (RKF) [56]. This was performed by dividing the estimated datasets into *K* = 10 blocks in various ways, with *N* = 10. For each of the *N* variants of partitions, the K-blocks were filled with different samples, resulting in a uniform distribution of classes. Sets of samples were created based on K-blocks for training and validating the classifier, with each K-block being validated once and the remaining *K* – 1 = 9 being used in training.

The classifier hyperparameters were then selected at the maximum average accuracy achieved on the validation set. K-block cross-validation allows for the selection of hyperparameter values that do not require retraining the model because many training and validation sets are used. Due to the optimization of hyperparameters on a fixed set of samples, it is possible that the average cross-validation accuracy is too optimistic. Consequently, after determining the optimal hyperparameters, the next step was taken. During the second stage, optimal values of hyperparameters were used and cross-validation was performed on other *N* = 30 partitions divided into *K* = 10 blocks, which was different from the first stage. Classification accuracy was measured based on the average *A*_RKF_ accuracy across the new partitions.

## 3. Experimental Results and Discussion

In this section, we present the results of assessing classification accuracy using all features, one signal type, all channels, one channel, and one feature.

### 3.1. Classification Accuracy Using One Method for Calculating the Entropy

**Figure 3:**
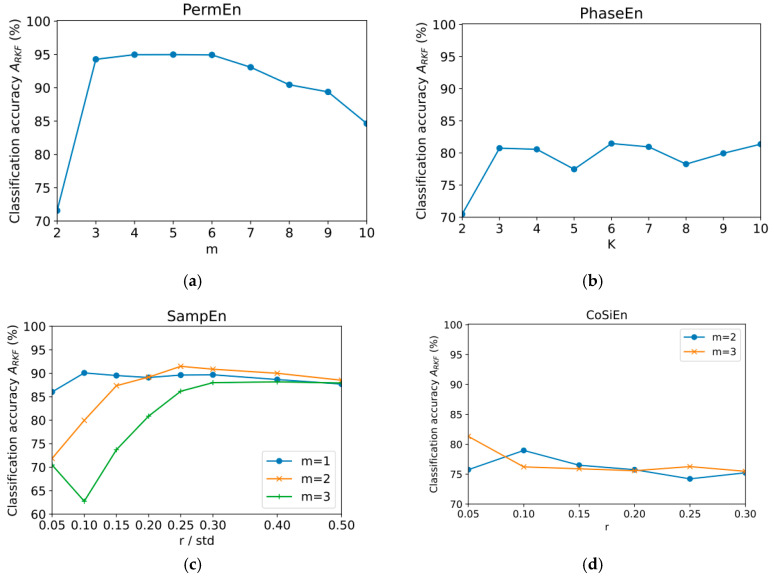

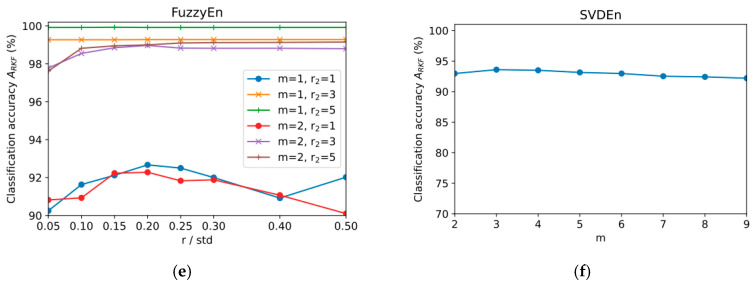
Dependence of classification accuracy *A*_RKF_ on entropy parameters using all 126 features for PermEn (**a**), PhaseEn (**b**), SampEn (**c**), CoSiEn (**d**), FuzzyEn (**e**), and SVDEn (**f**).

In both NC and PD, the entropy feature was computed using all nine types of input EEG data (original signal and eight reconstructed signals based on detail and approximation coefficients) across 14 channels (126 features in total). A model was developed to categorize NC and PD based on the features extracted from NC and PD pairs. Based on PermEn, SampEn, CoSiEn, FuzzyEn, PhaseEn, BubbleEn, and SVDEn, Figure 3 shows the classification accuracy (*A*_RKF_) of each entropy feature with different hyperparameters. These entropy features were computed with varying hyperparameter values in this study. Using five non-overlapping segments of 40 subjects (20 PDs and 20 NCs), we extracted entropy features from 200 datasets. In this task, the optimal parameters for each of the entropy calculations were determined.

The best classification result *A*_RKF_ = 99.9% was demonstrated for FuzzyEn with parameters (*m* = 1, *r* = 0.15 × std, *r*_2_ = 5). The influence of the r parameter in this case is insignificant. It was observed that the *A*_RKF_ value increases as the r_2_ parameter increases from 1 to 5. The next most accurate entropy method was AttnEn (*A*_RKF_ = 97.9%). This method has no hyperparameters. Acceptable accuracy was achieved for PermEn (*A*_RKF_ = 95% for m = 5) and SVDEn (*A*_RKF_ = 93.6% for *m* = 3). Both curves have a maximum at intermediate values of the m parameter. The worst results were obtained using the SampEn (*A*_RKF_ = 91.5% for *m* = 2, *r* = 0.25 × std), PhaseEn (*A*_RKF_ = 81.5% for *K* = 6), and CoSiEn (*A*_RKF_ = 81.3% for *m* = 3, *r* = 0.05) methods.

### 3.2. Classification Accuracy Using One Type of Signal

**Figure 4:**
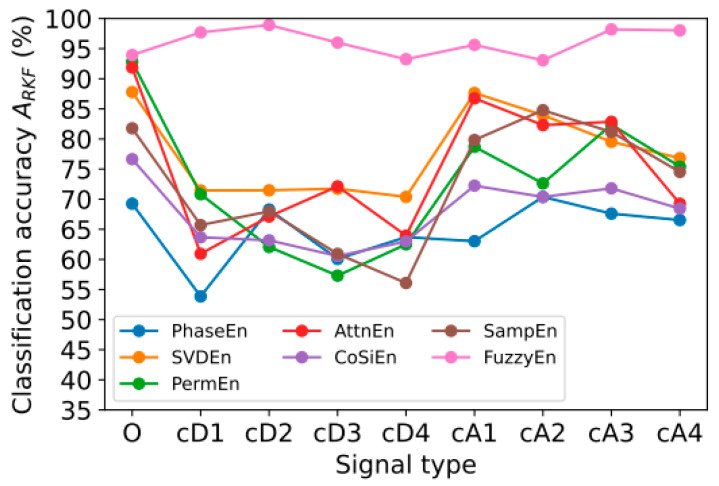
Dependence of classification accuracy *A*_RKF_ on signal type for different entropy calculation methods: PhaseEn (*K* = 6), SVDEn (*m* = 3), PermEn (*m* = 5), AttnEn, CoSiEn (*m* = 3, *r* = 0.05), SampEn (*m* = 2, *r* = 0.25 × std), and FuzzyEn (*m* = 1, *r* = 0.15 × std, *r*_2_ = 5).

Furthermore, we wished to identify which type of EEG data is most effective among the nine types of data, as described in Section 2.2, based on different entropy measures. Through this investigation, the computational complexity (memory and computation time) of the proposed PD diagnosis system can be reduced. This section presents the results of calculating classification accuracy *A*_RKF_ using each type of nine signals (O, cA1–cA4, cD1–cD4) for each of the 14 channels (14 features in total). The values of the optimal entropy parameters correspond to those presented in Section 3.1. Figure 4 shows the dependence of *A*_RKF_ on the type of signal.

According to the experimental results, FuzzyEn has higher accuracy than other types of entropy features for all types of signals. According to the presented data, it can be noted that the use of only one type of signal (14 features) generally reduces the accuracy of the *A*_RKF_ classification compared to using all 126 features. When using FuzzyEn, the *A*_RKF_ value had high values for the following signals: cD2 (*A*_RKF_ = 98.9%), cA3 (*A*_RKF_ = 98.2%), cA4(*A*_RKF_ = 98%). For other entropies, high *A*_RKF_ values were observed for signals O, cA1, cA2, cA3, cA4. Perhaps this is due to the presence of a low-frequency component in the range from 0 to 4 Hz in these signals, namely O (0–64 Hz), cA1 (0–32 Hz), cA2 (0–16 Hz), cA3 (0–8 Hz), and cA4 (0–4 Hz), while cD1 (32–64 Hz), cD2 (16–32 Hz), cD3 (8–16 Hz), and cD4 (4–8 Hz) signals contain higher frequency components. Low-frequency rhythms (delta and theta) are usually prominent while the eye is closed and in a resting state compared to waking and alert states (while the eye is open and focused). People with neurological disorders, particularly those with delta and theta rhythms, tend to have these rhythms dominate more than healthy individuals. Due to this, low-frequency rhythms (alpha to gamma) are more accurate in diagnosing Parkinson’s disease than high-frequency rhythms.

The decrease in accuracy when using only one type of signal is quite significant: classification error *E*_RKF_ = 1 − *A*_RKF_ increased by 11 times compared to the result achieved when using all features (Section 3.1). Thus, the use of one frequency range is not enough to achieve maximum classification accuracy *A*_RKF_ = 99.9%.

### 3.3. Classification Accuracy Using a Single Channel

**Figure 5:**
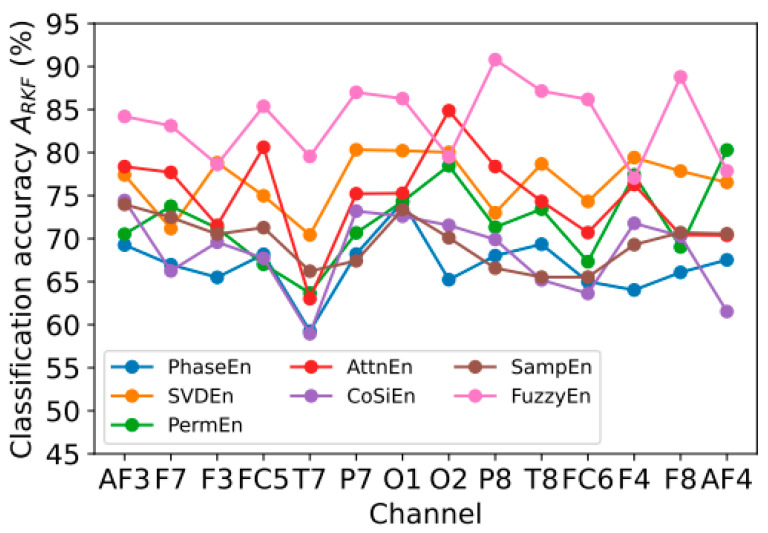
Dependence of classification accuracy *A*_RKF_ on the channel number for different entropy calculation methods: PhaseEn (*K* = 6), SVDEn (*m* = 3), PermEn (*m* = 5), AttnEn, CoSiEn (*m* = 3, *r* = 0.05), SampEn (*m* = 2, *r* = 0.25 × std), and FuzzyEn (*m* = 1, *r* = 0.15 × std, *r*_2_ = 5).

In this section, we present the results of classification accuracy *A*_RKF_ using all nine signal types (nine features in total) corresponding to one of the 14 channels (AF3, F7, F3, FC5, T7, P7, O1, O2, P8, T8, FC6, F4, F8, AF4). The values of the optimal entropy parameters are specified in Section 3.1. In Figure 5, *A*_RKF_ is shown in relation to the channel number.

Analyzing the results presented in Figure 5, it can be noted that the highest *A*_RKF_ value for most channels was obtained using FuzzyEn for the P8 (*A*_RKF_ = 90.8%) and F8 (*A*_RKF_ = 88.8%) channels. It is not possible to find pronounced dependencies that are repeated for all entropies. The classification accuracy obtained when using one channel is significantly reduced compared to the results achieved when using all channels: minimum classification error *E*_RKF_ increases by ~8 times when using one channel and one type of signal (Section 3.2) and 92 times when using all signals and all channels (Section 3.1). This suggests the need to use multichannel EEG measurement devices to maximize accuracy.

### 3.4. Classification Accuracy Using One Feature

In Section 3.2 and Section 3.3, reduced datasets with fourteen (one signal type) and nine (one channel) features were used; however, Figure 4 and Figure 5 show that classification accuracy varies significantly across different channels and signal types (frequency bands). At the same time, when analyzing these two criteria, we cannot determine the most informative combinations of channels and frequency ranges.

This section presents the results of using one feature (one type of signal for one channel). In this case, the FuzzyEn method, which produced the best accuracy estimate in Section 3.2 and Section 3.3, will be used, with the parameters *m* = 1, *r* = 0.15 × std, *r*_2_ = 5. The graphs are grouped by signal types and are divided into two groups:

1. Group 1 consists of signals based on detail wavelet coefficients, as follows: cD1 (32–64 Hz), cD2 (16–32 Hz), cD3 (8–16 Hz), and cD4 (4–8 Hz);
2. Group 2 consists of the original signal and signals based on approximation wavelet coefficients, as follows: O (0–64 Hz), cA1 (0–32 Hz), cA2 (0–16 Hz), cA3 (0–8 Hz), and cA4 (0–4 Hz).

**Figure 6:**
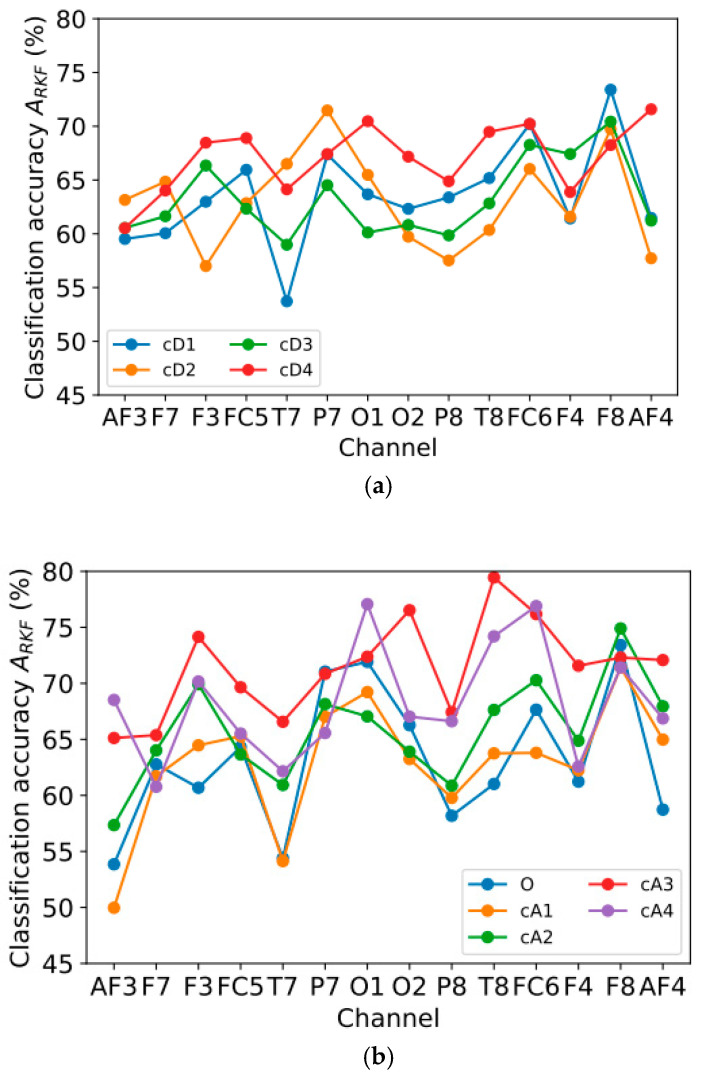
Dependence of classification accuracy *A*_RKF_ on channel number for FuzzyEn method (*m* = 1, *r* = 0.15 × std, *r*_2_ = 5), grouped by signal types: (**a**) cD1, cD2, cD3, cD4; (**b**) O, cA1, cA2, cA3, cA4.

The most informative frequency range for the first group (Figure 6a) is cD4 (4–8 Hz), for which the average value of *A*_RKF_ (*A*_RKF_mean_) is equal to 67.1%, while for the rest of the frequency ranges, *A*_RKF_mean_ ~63%. Among the signals in the second group (Figure 6b), the most informative is cA3 (0–8 Hz), with an average value of *A*_RKF_mean_ = 71.4%, while signals with the presence of higher-frequency components show lower values of *A*_RKF_mean_: 63.2% for O (0–64 Hz), 62.9% for cA1 (0–32 Hz), and 65.8% for cA2 (0–16 Hz). The lower accuracy of *A*_RKF_mean_ = 68.2% for cA4 (0–4 Hz) may indicate that the 4–8 Hz range is needed to improve signal classification accuracy. The highest classification accuracy by one feature was obtained for the T8 channel and the cA3 signal: *A*_RKF_ = 79.5%.

To determine the most informative combinations of channels and frequency ranges, Table 2 was compile. It contains 15 combinations of channel and signal type with the highest *A*_RKF_ value from those presented in Figure 6a,b. It can be noted that for most of the channels presented in the table (T8, O2, FC6, F3, AF4), only the low-frequency components of the original signal are the most informative, namely cA3 (0–8 Hz), cA4 (0–4 Hz), and cD4 (4–8 Hz), while for channels F8 and O1, signals with high-frequency components are also informative: O (0–64 Hz) and cD1 (32–64 Hz). It is also worth noting that most of the channels that give the best results were located in the right hemisphere of the head.

According to our knowledge, there are no earlier studies that examine the impact of specific regions or specific hemispheres on PD diagnosis using rest-state EEG signals. As a result of the proposed entropy-based PD diagnosis methodology, right hemisphere channels showed a significant difference compared to left hemisphere channels in terms of the following criteria: (a) limited number of PD subject data, clinical history of the patients, and progression of PD in the subjects; (b) limited number of channels (14 channels); and (c) proposed methodology of entropy features and machine learning-based diagnosis. No specific region in the brain has been studied in the literature on diagnosing PD due to the lack of valid scientific evidence. By conducting the experiment on another PD dataset with a larger number of subjects with a higher number of EEG channels, we could justify or test our proposed conclusion in the future.

## 4. Model Optimization

**Figure 7:**
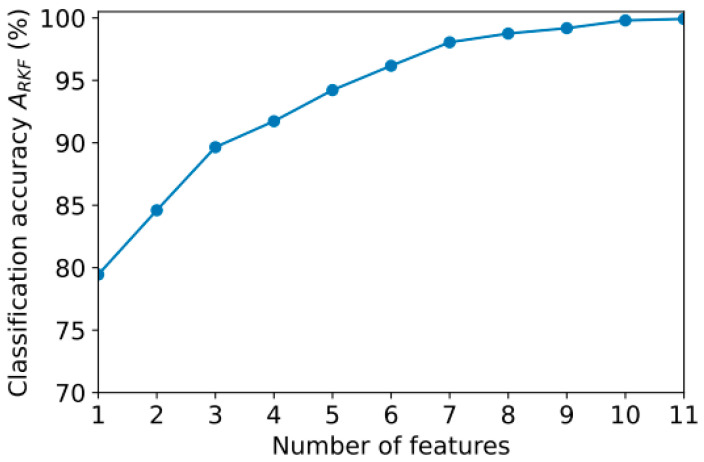
Dependence of classification accuracy *A*_RKF_ on the number of features.

Section 3.4 showed that different types of signals perform best on different channels. A high classification accuracy can be achieved with a minimum number of features, which appears to be an interesting goal. We examined how the accuracy of *A*_RKF_ changes with the number of features computed using FuzzyEn (Section 3.4). In order to do this, we used an iterative approach in which only the first feature gave the maximum value of A_RKF_. Next, the *A*_RKF_ value was calculated for the combination of two features. The evaluation procedure was repeated with one more of the remaining features added. Figure 7 illustrates the dependence of *A*_RKF_ on feature numbers.

With 11 features, classification accuracy *A*_RKF_ is 99.9%, which is the same as that achieved using all 126 features. By minimizing the number of features, it is possible to reduce the computational costs of classification and use lower-performance devices for analysis, such as peripheral IoT devices or embedded analytical modules in EEG signal measurement devices.

**Figure 8:**
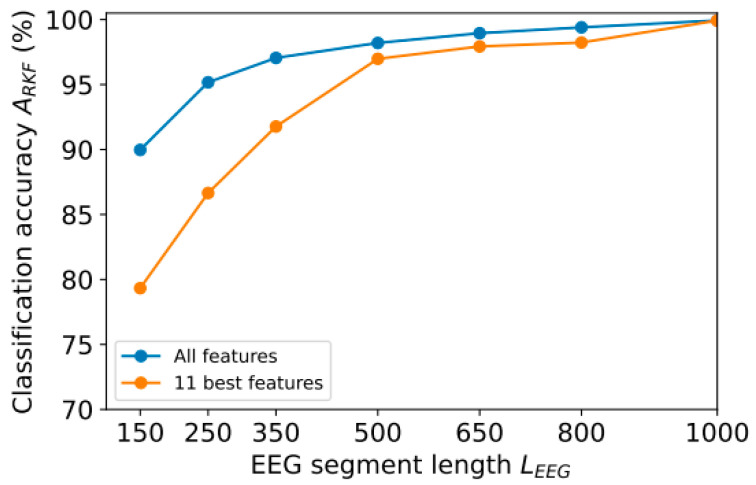
Dependence of classification accuracy *A*_RKF_ on segment length *L*_EEG_.

The length of the EEG segment (*L*_EEG_) can also be reduced to reduce the amount of data to be processed. In Section 3, we used segments with 1000 counts (~7.8 s). However, it is possible to shorten this length in order to speed up calculations. We achieved this by reducing the most resource-intensive part of the analysis—the calculation of FuzzyEn. Another part of the time is spent filtering the signal using wavelet methods. According to Figure 8, *A*_RKF_ accuracy depends on the number of *L*_EEG_ readings when using all 126 features (see Section 3.1) or the 11 most informative ones (this section).

The segment length *L*_EEG_ of 1000 samples provides a high classification accuracy of 99.9% for both 11 and 126 features. As segment length *L*_EEG_ decreases, classification accuracy *A*_RKF_ also decreases, but less intensely for 126 features than for 11. For example, a decrease in length even by 20% (up to *L*_EEG_ = 800) led to a decrease in accuracy to 99.4% for 126 features and to 98.2% for 11 features. Thus, *E*_RKF_ error increased by 6 times for 126 features and by 18 times for 11 features.

Since the main idea of reducing computational costs is to reduce computation time, we compared the computation time of one segment (calculation of entropy features and classification by the trained model) for different segment lengths *L*_EEG_ and different numbers of features. The calculations were performed on a desktop computer with an Intel i5-7200U (2.5 GHz) processor and 8 GB of RAM.

**Figure 9:**
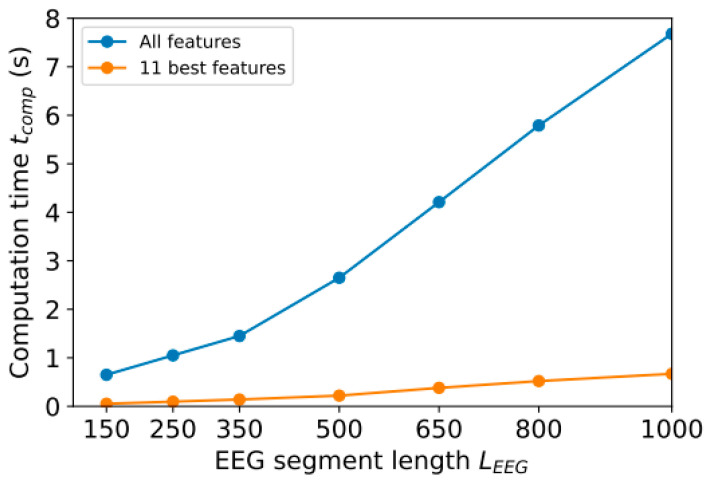
Dependence of computation time *t*_comp_ on segment length *L*_EEG_.

With more than 350 samples, computation time *t*_comp_ depends linearly on segment length *L*_EEG_, since most of the time is spent calculating entropy features. It took approximately 0.06 s to calculate one feature with a length of *L*_EEG_ = 1000. In Figure 9, it can be observed that by reducing the number of features, calculation time can be significantly reduced (for example, with *L*_EEG_ = 1000, calculation time varies by 11 times) while maintaining a low classification error (see Figure 8). The reduction in segment length does not significantly improve calculation speed (for example, the speed difference between *L*_EEG_ = 1000 and *L*_EEG_ = 800 is only 25%), but significantly increases classification error E_RKF_.

## 5. Future Work: Smart IoT Environment Concept for Patient Health Monitoring

**Figure 10:**
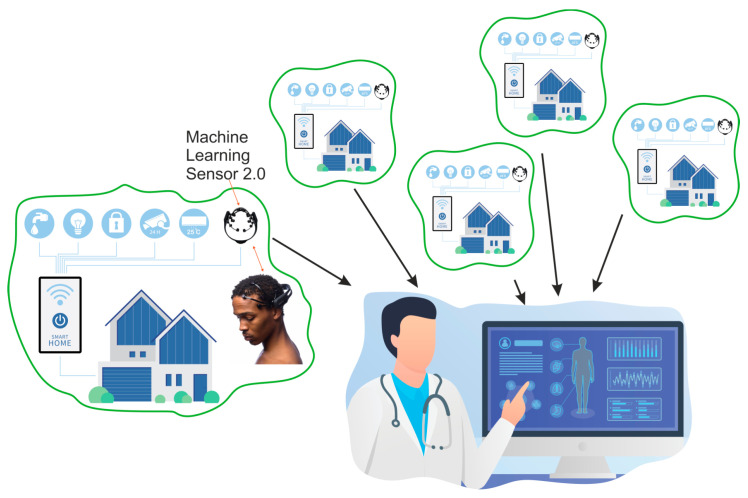
The concept of a smart IoT environment that can continuously monitor Parkinson’s disease patients.

Based on the results presented in Section 3, we conclude that entropy features can be used to analyze EEG signals in order to effectively diagnose PD patients. Let us present the idea of a smart IoT environment that continuously monitors the patient’s condition at home (Figure 10). Such a smart IoT environment collects and analyzes a wide array of information in real-time using ML sensors in edge IoT devices. The results are then presented to both the patient and the attending physician through remote, authorized access to the data. The latter is especially important if the treatment takes place at home rather than in a medical facility [57]. An attending physician can intervene quickly if a patient’s condition deteriorates, which the patient himself/herself may not be aware of due to the deterioration in cognitive functions. This approach enhances human resilience to PD, making everyday life more comfortable and easier.

**Figure 11:**
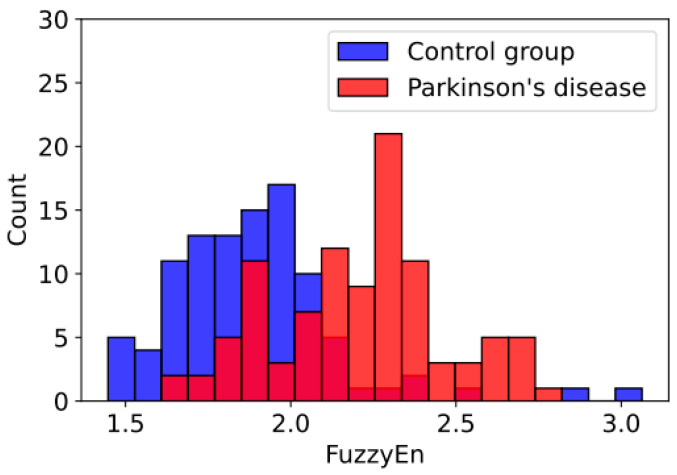
Histogram of distribution of FuzzyEn values for signal cA3 of channel T8.

According to the concept of personal medicine [8], the constant monitoring of disease and identifying the best treatment method for everyone are important elements of care. The previous sections discussed the classification of EEG signals used to diagnose Parkinson’s disease. FuzzyEn-based features, however, can be used as a tool to assess the current state of a disease. Histograms of entropy values (cA3 for channel T8) for people with Parkinson’s disease and healthy controls are shown in Figure 11. Based on the results presented, the presence of disease is associated with more chaotic EEG signals in most patients. Based on the dynamics of the change in entropy value, it is possible to track the improvement or deterioration of the clinical picture for each individual patient using several combinations of signal type and channel as indicators. As entropy increases, one can speak of deterioration in the patient’s condition, and as it decreases, one can speak of improvement. As a result of the variability in values within the dataset under study, the absolute value of entropy cannot serve as an unambiguous indicator of disease severity. The effectiveness of an individual treatment method can also be assessed based on how much entropy has decreased over time compared with control indicators. Thus, the results shown in Figure 11 can be expanded with additional studies to identify the connection between changes in FuzzyEn values of EEG signals and the degree of progression of PD using continuous monitoring with the proposed IoMT system.

The optimization of information processing processes is an important step in developing IoT environments and low-performance sensors that monitor PD patients’ health status. Due to their limited computing capabilities and small amounts of RAM, IoT devices and gateways need to reduce their volume to speed up data processing. An IoMT network is capable of continuously monitoring physiological parameter changes in humans by using machine learning (ML) models trained on smart sensors [12,58,59]. Physiological or biomedical sensors that are placed on the patient’s body (wearable sensors) measure different types of physiological responses, including heart rate, blood pressure, skin electrical conductivity, oxygen saturation, heart electrical activity, electroencephalograms (EEGs), etc. [60]. Additionally, some sensors can be placed in the room where the patient is located to monitor their movement patterns, gait, physical activity, etc. [61,62]. In addition to transforming the hardware designs of traditional sensor systems using ML techniques, artificial intelligence sensors (or smart sensors) can also be designed holistically based on ML methods [63] and machine learning algorithms [64,65]. A further development of the ML sensor paradigm was achieved by Warden et al. [59] and Matthew Stewart [58], where the authors introduced the terms Sensors 1.0 and Sensors 2.0. Sensors 2.0 involve both a sensor and a machine learning module integrated into one device.

In Section 4, we showed that only 11 features are sufficient to identify PD with a classification accuracy *A*_RKF_ of ~99.9%. For future research, it is possible to propose the development of a type of Sensor 2.0 which will be implemented in the real device (wireless headset) (Figure 10). EEG signals will be input into the model, and the output will be the degree of disease development. This may be part of a smart IoT environment for patient health monitoring. To implement the EEG signal classification methods proposed in this work, it is proposed that Raspberry Pi Zero W be used.

The third direction of research could be the fusing of information from EEG devices and an IoT video camera. Continuous monitoring of the patient’s condition could include regular (e.g., weekly) EEG measurements at rest and continuous monitoring of motor activity using video surveillance. By analyzing the video image, it would be possible to identify specific motor activity disorders characteristic of Parkinson’s disease. Both the patient and his/her attending physician would be able to monitor the patient’s condition objectively based on the analysis results. Interaction between the smart IoT environment and a medical information system could be achieved through network interaction. This would be especially relevant to remote northern regions with low population density and long distances to medical institutions with the necessary infrastructure. Additionally, it would reduce the burden on medical facilities and reduce the cost and time of transporting patients.

## 6. Conclusions

This study proposes a novel ML model based on EEG entropy features for PD diagnosis and monitoring in smart IoT environments. We investigated the most effective entropy method to calculate EEG entropy features. We found that fuzzy entropy performed well in detecting and monitoring Parkinson’s disease. EEG signals with low frequencies (0–4 Hz) contributed the most to high classification accuracy, and we identified the most prominent EEG signal frequency range. Additionally, the most informative signals were received primarily from the right hemisphere of the head (F8, P8, T8, FC6). A combination of signal frequency range and channels was selected to accurately diagnose PD with only 11 features achieving a classification accuracy *A*_RKF_ of ~99.9%, while reducing data processing time by ~11 times. A study of the dependence of classification accuracy *A*_RKF_ on the length of EEG segments (*L*_EEG_) showed a significant decrease in *A*_RKF_ with a decrease in *L*_EEG_: from 99.9% for *L*_EEG_ = 1000 to 98.3% for *L*_EEG_ = 800 when using the 11 best features. At the same time, decreasing the value of *L*_EEG_ only slightly reduced computation time, so this approach does not make much practical sense. This also shows the limitations of the method: to obtain a high classification accuracy, it is necessary to use long segments of the EEG signal (1000 samples or ~7.8 s). An optimized model with a small number of features, reducing computational costs, could be used in low-performance devices, and so would be applicable for smart IoT environments with ML sensors.

## Figures and Tables

**Table 1:** Ranges of parameters used to create entropy features.

Entropy Name	Parameter Range
SVDEn	order *m* = 2…10, *delay* = 1
PermEn	order *m* = 2…10, *delay* = 1
SampEn	order *m* = 1…3, tolerance *r* = 0.05…0.5 × std
CoSiEn	order *m* = 2…3, tolerance *r* = 0.05…0.5
FuzzyEn	order *m* = 1…2, tolerance *r* = 0.05…0.5 × std, exponent membership function of order *r*_2_ = 1…5
PhaseEn	*K* = 2…10
AttnEn	no parameters

**Table 2:** Combinations of channels and signal type that give the highest *A*_RKF_ value.

Channel	Signal Type	*A*_RKF_, %
T8	cA3	79.5
O1	cA4	77.1
FC6	cA4	76.9
O2	cA3	76.5
FC6	cA3	76.2
F8	cA2	74.9
T8	cA4	74.2
F3	cA3	74.2
F8	O	73.4
F8	cD1	73.4
O1	cA3	72.4
F8	cA3	72.3
AF4	cA3	72.1
O1	O	71.9
AF4	cD4	71.6

## Data Availability

The data are not publicly available due to their containing information that could compromise the privacy of research participants. Data requests can be sent to Murugappan Murugappan through his email m.murugappan@kcst.edu.kw.
